# Species diversity and DNA barcode library of freshwater Molluscs of South Caucasus

**DOI:** 10.3897/BDJ.10.e84887

**Published:** 2022-09-13

**Authors:** Ani Bikashvili, Nino Kachlishvili, Bella Japoshvili, Levan Mumladze

**Affiliations:** 1 Institute of Zoology, Ilia State University, Tbilisi, Georgia Institute of Zoology, Ilia State University Tbilisi Georgia

**Keywords:** South Caucasus, DNA barcode library

## Abstract

This study provides the first attempt to investigate the molecular diversity of South Caucasian freshwater molluscs (Mollusca, Gastropoda) and lay down the first bricks to build up a DNA-barcode library. In total, 289 COI barcode sequences were obtained from 33 morpho-species belonging to 24 molluscan genera and 10 families that represent nearly 30% of known freshwater molluscan diversity of the South Caucasus region. DNA barcodes were analysed by means of the Barcode Index Number (BIN) and the other tools available in BOLD Systems. Results showed that the knowledge of freshwater molluscs diversity in the South Caucasus is far from comprehensive. For the studied 33 morpho-species, 289 barcodes were clustered into 40 BINs, from which unique BINs were defined for 12 species and five species were characterised with more than a single BIN. From the studied taxa, 60% were characterised larger than 2.2% sequence divergence indicating high genetic variation or cryptic diversity. Within our limited taxonomic coverage, we found one new species for the Republic of Georgia (*Galbaschirazensis*) and at least three undescribed species belonging to the genera *Stagnicola*, *Segmentina* and *Anisus*. Uniqueness and high molecular diversity of the studied species emphasise the need for further intensive morphological and molecular investigations of the South Caucasian freshwater molluscan fauna.

## Introduction

Under increasing anthropogenic pressure, the conservation of freshwater biodiversity and maintaining freshwater ecosystem functioning remain two of the most critical challenges for the 21st century’s world (Butchart et al. 2010, Hoffmann et al. 2010). A sufficient knowledge of the species diversity and distribution of freshwater taxa is crucial for understanding the needs and implementation of conservation measures to save species and maintain ecosystem integrity (Collier et al. 2016). Freshwater molluscs constitute a diverse and functionally important component of freshwater communities (Runck 2007, Strong et al. 2007) inhabiting a wide range of freshwater habitats (Dillon 2000, Strong et al. 2007) and, at the same time, are the most vulnerable taxa amongst freshwater inhabitants (Cuttelod et al. 2011). Accurate biodiversity information on freshwater molluscs is often missing, especially in species-rich and economically poorly devolved parts of the world, hindering effective management and conservation activities. A good example is the Caucasus biodiversity hot-spot where, in spite of the recent advancements (e.g. Vinarski et al. 2014, Grego et al. 2020, Chertoprud et al. 2020, Chertoprud et al. 2021, Bikashvili et al. 2021, Neiber et al. 2021), the knowledge about the diversity and distribution of freshwater molluscs is still far from being comprehensive (Mumladze et al. 2019, Mumladze et al. 2020). Most probably this is due to the absence of local taxonomic expertise during the last 50 years.

Recent developments of DNA barcoding technology helped significantly to revive and advance the biodiversity inventory and monitoring at an unprecedented rate (Waugh 2007, Trivedi et al. 2016). DNA barcoding proved to be an effective tool in helping taxonomists to distinguish taxa and even confidently solve the taxonomic problems, especially when traditional (morphology - based) methods alone are failing (Hebert et al. 2003, Hajibabaei et al. 2006, Goldstein and DeSalle 2011, Sheth and Thaker 2017). Perhaps more importantly, DNA barcoding triggers even non-taxonomists and the young generation to put effort into biodiversity investigation (Packer et al. 2009, Ellis et al. 2010, Ebach 2011). For instance, in Georgia, a number of research projects have been conducted very recently investigating the freshwater biodiversity, including or exclusively being based on DNA barcoding approaches conducted by experienced and amateur scientists (Grego et al. 2020, Epitashvili et al. 2020, Japoshvili et al. 2020, Bikashvili et al. 2021). In addition, DNA barcoding (and in particular environmental DNA or eDNA meta-barcoding) is a promising tool in fast, non-invasive and cost-effective means for biodiversity inventory/monitoring (Thomsen et al. 2012, Carew et al. 2013, Thomsen and Willerslev 2015). However, in order to make DNA barcoding approaches useful tools, it is essential to build barcode reference libraries against which newly-obtained barcodes can be compared (Leese et al. 2018, Weigand et al. 2019). A barcode reference library is basically a data infrastructure that requires a routine input from both taxonomic and molecular experts. Currently, the largest reference library is available in BOLD systems (http://www.boldsystems.org) which is, on the other hand, less effective when dealing with taxa from poorly-investigated areas (Weigand et al. 2019). For instance, for the Caucasus region, barcode information is lacking for a great deal of taxa, including freshwater molluscs. In the present publication, we provide a first stage of an ongoing project that aims to build a DNA barcode reference library for South Caucasian freshwater molluscs within the framework of the Caucasus Barcode of Life initiative (https://ggbc.eu). In particular, the aim of the given study was to: (1) generate COI barcode sequences for a part of the freshwater molluscan taxa known for the region, (2) investigate within vs. between species sequence variation, (3) identify gaps in species-level taxonomic knowledge of freshwater molluscs and (4) develop subsequent research agenda.

## Materials and methods

### Sample Collection

**Figure 1.**
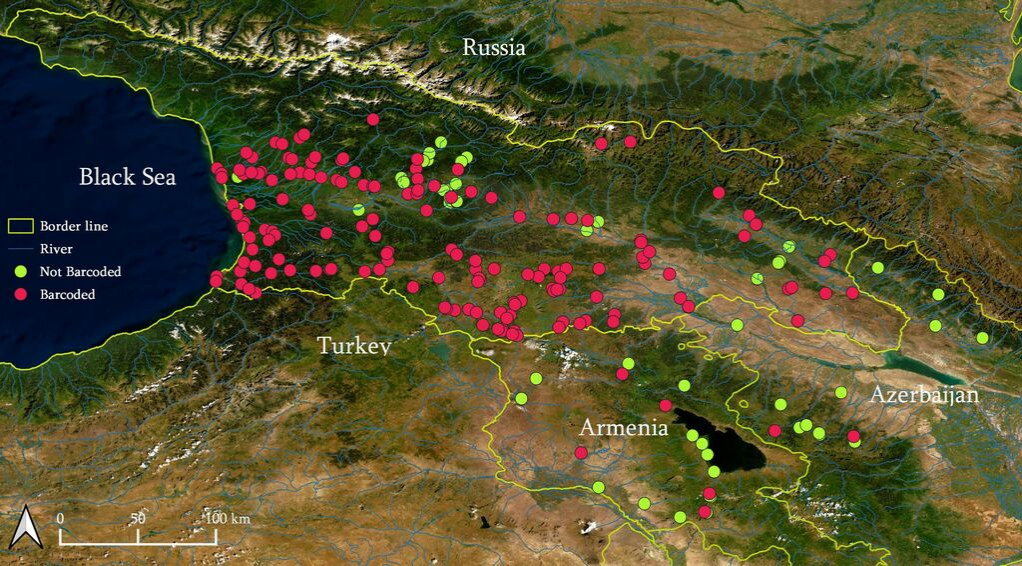
Map of collection localities for freshwater molluscs in the present study. The red dots correspond to the localities from where one or more specimens/species were submitted to barcoding, while the yellow dots correspond to localities from where the specimens are still waiting for genetic investigation.

Sample collection campaigns were carried out from 2015 to 2021 across the various regions of Georgia (and also, to a lesser extent, in Armenia and Azerbaijan during 2019) (Fig. 1). The territory of Georgia is very rich with natural lotic and lentic water bodies and is, thus, hard to sample exhaustively. To provide a representative sampling scheme, we planned field collection trips for every municipal region of Georgia and, during each collection trip, we sampled as many independent water bodies as possible. For each geographic locality, we tried to do exhaustive sampling by checking all kinds of available habitats including river banks, springs (including subterranean), channels, lake littorals, mires and temporal water bodies, as well as subterranean habitats (caves and springs). Specimens were collected by sieving substrates from different types of microhabitats and also directly from the surfaces of water plants and fallen leaves, stones and sink logs. In addition and whenever possible, bottoms of lotic/lentic habitats were inspected with glass bottom viewing boxes for mussels of the family Unionidae. In case of Armenia and Azerbaijan, only a single (though long distance) per-country field collecting trip was arranged with the same field collecting principles. Samples were immediately preserved in 96% ethanol after collection. Sorting and taxonomic identification of individuals was conducted using the keys of Jackiewicz (1998), Glöer (2002), Soldatenko and Starobogatov (2004), Welter-Schultes (2012), Piechocki and Wawrzyniak-Wydrowska (2016), Glöer (2019) and Vinarski et al. (2020).

One to ten specimens per morphologically defined species were selected for barcoding. In cases of genera - *Radix* and *Ancylus* for which the systematics of Caucasian taxa is not yet well understood, we took a larger number of specimens for each morpho-species. All selected specimens were first photographed according to BOLD standards (Milton et al. 2013) and, in the case of larger specimens, only a part of tissue was separated for DNA extraction, while, for small-bodied species (such as, for instance, *Ancylus* and most of Sphaeriidae), the soft body of the complete individuals was submitted for DNA extraction. Here we must note that the family Hydrobiidae is the single exception that was not studied within the framework of the given article. The reason is that these prosobranch molluscs, which were known with only a few species until very recently (i.e. 5 years ago), proved to be highly diverse in the Caucasus region (Grego et al. 2020, Chertoprud et al. 2020, Chertoprud et al. 2021), and are currently under intensive taxonomic investigation. Due to a large, yet undescribed species diversity, we omit them from the current article.

Collected materials/vouchers are deposited in the collection of the Institute of Zoology of Ilia State University, Tbilisi under the respective CaBOL identification numbers given in Suppl. material 1.

### DNA processing

Genomic DNA was extracted from tissue samples using the Quick-DNA™ Miniprep Plus Kit (Zymo Research) (for 25 mg tissue), Quick-DNA™ Miniprep Plus Kit (Zymo Research) (for 5 mg tissue) DNeasy Blood & Tissue Kits (Qiagen, Germany) according to the manufacturer’s instructions and the protocol proposed by Sokolov (2000) with slight modifications (Sauer and Hausdorf 2009). Partial sequences of cytochrome oxidase c subunits I (COI) were amplified by polymerase chain reaction (PCR) using the primer pair LCO1490-JJ and HCO2198-JJ (Astrin and Stüben 2008). Thermal conditions included denaturation at 95°C for 1 min, followed by the first cycle set (15 cycles): 94°C for 30 sec, annealing at 55°C for 1 min (−1°C per cycle) and extension at 72°C for 1:30 min. Second cycles set (25 cycles): 94°C for 35 sec, 45°C for 1 min, 72°C for 1:30 min, followed by 1 cycle at 72°C for 3 min and final extension step at 72°C for 5 min. In addition, shorter COI sequences were amplified using the Folmer et al. (1994) forward (LCO1490) and Kuhn’s reverse (LCO1491) primers (cited in Cordellier and Pfenninger 2008). PCR cycling conditions were adopted from Wethington and Lydeard (2007) and were comprised of an initial denaturation step: 94°C for 3 min, followed by 30 cycles at 94°C for 40 sec, annealing temperature at 48°C for 1 min, 72°C for 1 min and final extension step at 72° for 10 min. Resultant amplicons were visualised on 1% agarose gels using 3 μl of PCR product. The remaining PCR products were then completed using Big Dye Terminator v.3.1 (Applied Biosystems, Foster City, CA, USA) and run on an automated sequencer. Some of the PCR products were sequenced at Macrogen Europe Laboratory (Amsterdam, The Netherlands). Both DNA strands of the PCR product were sequenced.

### Data analyses

Sequences were edited in Geneious Pro v.7 (Drummond et al. 2011) to ensure the absence of indels and stop codons. Quality sequences (i.e. less than 1% base-pair ambiguity) were submitted to BOLD Systems (http://www.boldsystems.org) under the project acronym “GEOFM” including the specimen images, trace files and the rest of the metadata (Suppl. material 1). In addition, we ran a BOLD search for molluscan barcodes originating from the South Caucasus region and which were added to the “GEOFM” project under a dataset named “DS-FMOL” for part of the analyses.

Barcode Index Numbers (BIN) (Ratnasingham and Hebert 2013) were then automatically assigned to each newly-derived sequence by BOLD Systems v.4. That is a two-stage analysis where, at the first stage, an initial assignment of sequence to an Operational Taxonomic Unit (OTU) takes place, based on Refined Single Linkage Clustering (RESL) with a threshold of 2.2% sequence differences. In the second stage, graphical analyses (Markov clustering) are applied to OTUs. Which, in case of the existence of a clearly defined internal structure within OTU, can result in its split into two or more OTUs in spite of smaller (i.e. less than 2.2%) sequence divergence between OTUs (Ratnasingham and Hebert 2013). RESL algorithm and ABGD (Automatic Barcode Gap Discovery - Puillandre et al. 2011) were further employed to generate OTUs and cluster histograms via BOLD Systems.

## Results

In total, 289 COI barcode sequences were obtained and uploaded in the “GEOFM” BOLD project, representing 33 species from 24 molluscan genera from 10 families. Prior to the present study, there were 47 freshwater molluscs COI barcode sequences available in the BOLD Systems (from the study area) including 11 sequences from an unpublished project within the “DNAqua-Net” COST Action (Leese et al. 2016) (*Viviparuscostae* (2), *Theodoxusfluviatilis* (2), *Bithyniatentaculata* (1), *Corbiculafluminalis* (2), *Anisus* sp. (1), *Planorbisplanorbis* (1), *Musculiumlacustre* (1), *Euglesa* sp. (1)) and 36 sequences mined from GenBank (11 sequences of *Ancylus* spp. (Bikashvili et al. 2021), 23 sequences of Hydrobiidae spp. (Grego et al. 2017, Grego et al. 2020) and a single sequence of *Melanopsismingrelica* (Neiber and Glaubrecht 2019) and *Radixeuphratica* (Aksenova et al. 2019).

The average fragment length of COI barcodes in the “DS-FMOL” dataset (combining “GEOFM” project plus pre-existing barcodes) was 534 bp (min: 409 bp and max: 658 bp). Nucleotide base frequencies were: A-25.4%, G-18.4, C-14.4%, T-41.8%) - similar to reported frequencies for molluscs (e.g. Weigand et al. 2011), while GC content equal to 32.8% was lowest compared to results from other molluscan studies (35.8% and 36.9% from Kumar et al. (2015) and Layton et al. (2014), respectively).

**Figure 2.**
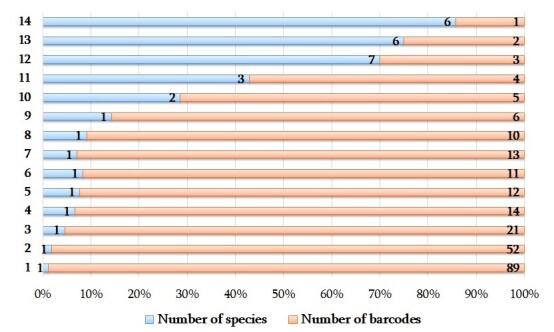
Ranking of species according to the number of barcodes.

The families Planorbidae and Lymnaeidae are represented by the highest number of barcodes (116 and 99, respectively). The two families Unionidae and Neritidae are represented each with 19 and 12 barcodes, respectively. The two families Cyrenidae and Sphaeriidae are represented by an equal number of barcodes (each with 11 barcodes). The two families Physidae and Viviparidae are represented each with 10 and 5 barcodes, respectively and the family Melanopsidae and Acroloxidae by the lowest number of barcodes (three barcodes each). The most common genus was *Ancylus*, for which 93 barcodes (two species) were generated, followed by *Radix* and *Unio* (73 and 16 barcodes, respectively and three species for each of them). The 18 genera were represented by a single species, two genera with two species and a single genus by the four species. Of all species obtained, two species *Ancylus* sp. 2 and *Radixauricularia* were represented the highest number of barcodes (each with 89 and 52 , respectively), followed by *Radixeuphratica*, *Lymnaeastagnalis*, *Theodoxusfluviatilis*, *Corbiculafluminalis*, *Uniocrassus* and *Physellaacuta* (each with 21, 14, 12, 11, 13 and 10 barcodes, respectively). Most of the species are represented with less than 10 barcodes, including six species, with a single barcode (Fig. 2).

The BIN and RESL analyses resulted in 41 BINs united into 40 OTUs. In addition, 13 OTUs were also formed for 23 sequences (all belonging to Hydrobiidae and mined from GenBank) for which no BINs had been defined due to the small barcode size (less than 500 bp (Ratnasingham and Hebert 2013)). From the 41 BINs, 32 (78%) were concordant and nine (22%) were represented with singletons. Sequences (107) of 18 BINs (42%) are only known from the study area at the time of publishing.

Average within species divergence were 0.69 ± 0.0% (ranged from 0% to 4.1%) followed with divergence of 6.4 ± 0.0% within genera (ranged from 0 to 16.7%) and 17.8 ± 0.0% divergence within families (ranged from 10.42% to 21.9%).

In most cases, morphologically determined specimens (comprising 28 species) were matched with a single OTU/BIN cluster with intraspecific (or within BIN) sequence divergence of less than 2.2%. More than one BIN were found in five species-level taxa - *Planorbisplanorbis* (2 BINs), *Physellaacuta* (2 BINs), *Lymnaeastagnalis* (2 BINs), *Radixauricularia* (2 BINs) and *Radixeuphratica* (4 BINs) (Table 1).

## Discussion

South Caucasian freshwater molluscs (and all invertebrates in general) are still poorly known (Japoshvili et al. 2016, Mumladze et al. 2020). The check-list of freshwater molluscs species for the South Caucasus or any separate country within it is more than 50 years old and completely outdated (Zhadin 1952, Javelidze 1973, Akramowski 1976). While a number of papers have appeared during the last three decades providing information on the taxonomy and systematics of separate taxa (given below), only three articles have been published reporting the field research-based inventory results of all freshwater molluscs of a particular area: for Sevan Lake in Armenia (Mashkova et al. 2018), Javakheti region of Georgia - Bikashvili et al. (2021) and Kazbegi Municipality in Georgia - Neiber et al. (2021). Thus, it is clear that the current knowledge of freshwater molluscs species diversity and distribution in the South Caucasus region remains far from being comprehensive.

Within the current project, we were able to generate 298 new barcodes corresponding to 33 freshwater mollusc species-level taxa. Roughly, this is no more than 30% of the expected species number in the South Caucasus (based on Vinarski and Kantor 2016, Glöer 2019, Mumladze et al. 2019, Grego et al. 2020). Nearly all morphologically identified species were further validated with barcode data, while several species turned out to be mismatches with the BOLD taxonomy. This latter category includes pond-snail species of the family Lymnaeidae, ramshorn snails (family Planorbidae) and freshwater clams (family Sphaeriidae). While the aim of this article is not to deal with the systematics and taxonomy of species, in the following, we will revise each of the studied taxa and outline gaps in the knowledge deemed for further in-depth study.

Pond snails of the family Lymnaeidae are distributed worldwide (Correa et al. 2011). They are of major medical and veterinary importance since they act as vectors of parasites (Bargues et al. 2006, Medeiros et al. 2014). The morphological and anatomical plasticity amongst and within lymnaeid representatives remains challenging (Bargues and Mas-Coma 1997, Jackiewicz 1998, Pfenninger et al. 2006, Aksenova et al. 2018); however, recent large scale multi-marker molecular genetics and morpho-anatomical investigations refined species-level taxonomy at least for a part of taxa within this family (Aksenova et al. 2018, Vinarski et al. 2020). Unfortunately, only four sequences of a single species (*Radixauricularia*) were available for the whole south Caucasus (in particular from Armenia) at the time of the studies cited above. According to literature, there are at least six genera of two subfamilies distributed in the South Caucasus including *Ampullaceana*, *Peregriana*, *Radix* (all three from the subfamily Amphipepleinae), *Galba*, *Stagnicola* and *Lymnaea* (all three from the subfamily Lymnaeinae).

Amphipepleinae represents one of the most species-rich and taxonomically challenging groups. Morphologically identified species - *Ampullaceanalagotis* formed the unique BIN BOLD:AEN6567 with the divergence of 4.97% to the nearest neighbour (NN) BIN BOLD:ACI0501 that includes specimens of yet unresolved “*Radixzazurnensis*” from Russia (3) and China (32) (Aksenova et al. 2016). Thus, this species is represented in our database as *Ampullaceana* sp. awaiting further taxonomic clarification. In contrast, specimens identified as *Peregrianaperegra* (widely referred to as *Radixlabiata*) perfectly matched with BIN BOLD:AAD0368 (with a maximum intra-BIN distance 4.92%) representing the same species from western Palearctic.

The genus *Radix* turned out to be the most complex within the family Lymnaeidae. Based on morphology alone, we were able to confidently identify only *R.auricularia*, barcodes of which formed two separate BINs: 12 specimens were allocated to BIN BOLD:ACI2007 (with 2.88% divergence to NN, BOLD:AAD6712) and 40 specimens were formed under BIN BOLD:AAD6712 (2.88% divergence to NN BOLD: ACI2007). Both BINs seem to characterise geographically variable *R.auricularia* populations. Other unidentified specimens of *Radix* (22 in total) formed four unique BINs, including 17 Georgian specimens that were classified under the BIN BOLD:ADJ8863. With our specimens, this BIN includes specimens from Iraq, Iran, Uzbekistan and Russia and represents species *R.euphratica* (with NN BIN BOLD:AEI7975 (2.82% divergence) representing a single specimen of *R.euphratica* from Iran). Five other specimens of *Radix* sp. formed three different BINs, BOLD:ADK5204 (with 3.37% divergence to NN BIN, BOLD:ADJ8863), BOLD:ADK6106 (with 1.92% divergence to NN BIN BOLD:ADR3052) and BOLD:ADR3052 (with 1.92% divergence to NN, BIN BOLD:ADK6106). Due to its small within-BIN distances, specimens can be named as *R.euphratica* which was first mentioned from the Tbilisi Reservoir (voucher number Mlym68 (Russian Museum of Biodiversity Hotspots, Federal Center for Integrated Arctic Research of the Russian Academy of Sciences, Arkhangelsk, Russia) (Aksenova et al. 2019). Our research has shown that *R.euphratica* is widespread in Georgia (13 sampling points in this study).

**Figure 3.**
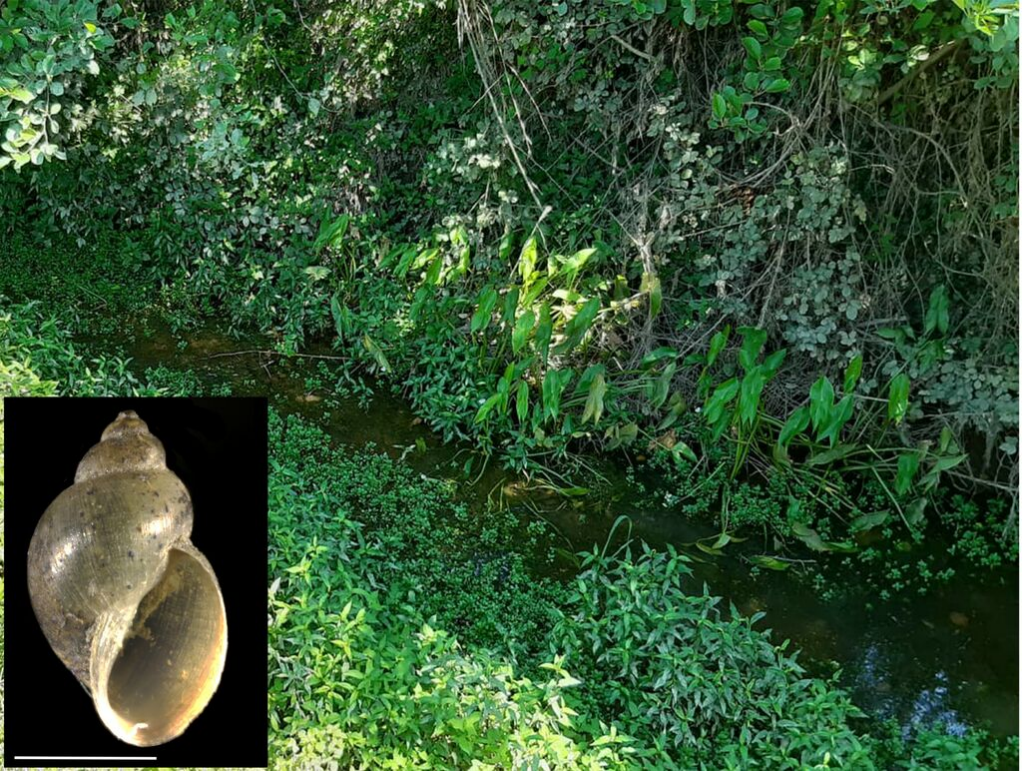
The shell of *Galbaschirazensis* and sampling location where specimens were collected: Orulu Village, Samegrelo – Zemo Svaneti Region, Georgia. Scale bar = 2 mm.

Subfamily Lymnaeinae includes three representative genera in South Caucasus each with a single species. *Galba* is characterised by high phenotypic plasticity and extremely uniform anatomical traits, which are often the reasons for species misidentification (Samadi et al. 2000, Standley et al. 2013). Three of our specimens of *Galbatruncatula* formed BIN BOLD:ABA2623 which represents the cluster of *G.truncatula* specimens from all over its distribution area. Distance to its NN BIN (BOLD:AAI7214) is 4.03% and is also named as *G.truncatula*. The single specimen (Samegrelo region, western Georgia) in our dataset (also morphologically identified as *G.truncatula*) clustered under BIN BOLD:AAY4012 comprising specimens of *Galbaschirazensis*. The NN (with 7.84% divergence) BIN is BOLD:ADR2784 includes the specimens of *Galbatruncatula* from Japan. A cryptic species - *G.schirazensis* was discovered relatively recently in different geographical regions throughout Europe, America and the Middle East, including Iran (Bargues et al. 2011). According to Kruglov (2005), *G.schirazensis* is already known from Azerbaijan - from a Caspian Sea Basin. For Georgia, it is a new country record. The specimens of *G.schirazensis* were collected from the western part of Georgia (Black Sea Basin), Orulu Village in Zugdidi District, Samegrelo – Zemo Svaneti Region (42.398926N, 41.739213E). In this location, specimens were found amongst vegetation in a permanent stream. The water was shallow and slow running (Fig. 3).

From the genus *Lymnaea* a single species – *L.stagnalis* is known. Our specimens of *L.stagnalis* formed two BINs. Eight specimens matched with BIN BOLD:AEN6037, for which only a single barcode was available from Ukraine. The NN BIN is BOLD:ACQ0092 with 2.43% divergence, includes specimens also belonging to *L.stagnalis*. The remaining six specimens formed the unique BIN (BOLD:AEM9638) with the NN BIN - BOLD:ACQ2679 (with 2.12% divergence) comprising specimens of *L.stagnalis*. Thus, in South Caucasus, at least two haplotypes of *L.stagnalis* occur, both in a mountainous Javakheti region (southern Georgia). The last genera in this subfamily is *Stagnicola* which is also represented with a single species (*S.palustris*) in South Caucasus. Only two specimens of *Stagnicola* were represented in our dataset forming the unique BIN - BOLD:AEN6388 which were diverged by 4.83% from NN BIN BOLD:ACV7473, representing the specimens of *S.turricula* from Poland. Most probably the genus *Stagnicola* in Georgia (and in South Caucasus) is not a *S.palustris* or the genus is represented with more than one species in the region. Thus, additional sampling and taxonomic investigation are required.

The Ramshorn snails of the family Planorbidae is the most diverse group of freshwater pulmonates inhabiting a wide range of freshwater habitats (Jørgensen et al. 2004, Albrecht et al. 2007). Understanding of relationships within the Planorbidae remains confused due to the extreme variability of anatomical and shell morphological traits (Baker 1945, Hubendick 1978). In South Caucasus, more than 15 species of Planorbidae are provisionally listed including the genera *Planorbis*, *Segmentina*, *Anisus*, *Hippeutis*, *Bathyomphalus*, *Gyraulus*, *Ancylus* and *Ferrissia* (Vinarski and Kantor 2016). For the current study, we obtained samples for seven out of eight genera, including the following morpho-species: *P.planorbis*, *S.nitida*, *A.leucostoma*, *G.albus*, *B.contortus*, *F.californica*, *Ancylus* sp. 1 and *Ancylus* sp. 2.

Seven specimens of *Planorbisplanorbis* formed two BINs - BOLD:AED0778 and BOLD:ADJ5964 diverged both from the same NN BIN (BOLD:ACS1294) with 3.4% and 2.1%, respectively. All three BINs are considered as *P.planorbis* in BOLD systems comprising the specimens from different regions of Europe and Middle East.

The genus *Segmentina* is taxonomically understudied. Some authors consider only a single *S.nitida* species within the genus (Falkner et al. 2001, Welter-Schultes 2012), while others (e.g. Kruglov and Soldatenko 1997) consider 14 separate species within the genus, including two species (*S.caucasica* and *S.malkae*) endemic to the north Caucasus. In this study, three specimens from South Caucasus (western Georgian lowlands) identified as *S.nitida* based on shell shape, formed the unique BIN BOLD:AEN3217 for which the NN BIN is BOLD:AAN3912 (with 11.89% divergence), comprising specimens of *Segmentina* sp. (52) and *S.nitida* (3) are from Poland, Sweden and Germany. This specimen apparently does not belong to *S.nitida* and is, instead, either a new species or does belong to one of those species indicated by Kruglov and Soldatenko (1997) for which no DNA sequences are available. Further study is required to solve the taxonomy of South Caucasian *Segmentina* spp.

Two representatives of the genus *Anisus* is known for South Caucasus (*A.leucostoma* and *A.spirorbis*) (Vinarski and Kantor 2016, Glöer 2019). Six specimens of *Anisus* in our dataset formed a unique BIN BOLD:AEC8114 which diverged from NN BIN BOLD:AAR3430 (*A.spirorbis* from Germany) by 8.58%. Thus, our specimens matched neither *A.spirorbis* nor *A.leucostoma* and most probably represent new, yet undescribed species.

The taxonomy of the genus of *Ancylus* is far from being resolved. For the Caucasus region, six species are indicated (Akramowski 1976, Soldatenko and Starobogatov 2004). For the present study, 104 specimens collected throughout Georgia and Armenia (that were initially identified as four morpho-species of *A.benoitianus*, *A.capuloides*, *A.major* and *Ancylus* sp. according to Soldatenko and Starobogatov 2004) were classified into two BINs. In particular, 12 specimens (*Ancylus* sp. 1) were defined under BIN BOLD:AEN7656 with 4.58% divergence to NN BOLD:AAD2028 and 92 specimens (*Ancylus* sp. 2) were defined under BIN BOLD:AAD2028 with 3.3% divergence to NN BOLD:ACZ3241. It is worth noting that neither of the above-mentioned BINs are properly named. The Caucasian *Ancylus* is characterised with a large number of lineages similar to those revealed in the Balkans (Albrecht et al. 2006) or in Germany (Weiss et al. 2018), thus reflecting the taxonomy of Soldatenko and Starobogatov (2004). However, overall genetic (and morpho-anatomical) differentiation might not be enough to delimit the species. Nonetheless, it is evident that Caucasian *Ancylus* is represented with a rather unique complex of lineages deserving further in-depth integrative taxonomic investigation.

The remaining Planorbidae species – *Ferrissiacalifornica*, *Gyraulusalbus* and *Bathyomphaluscontortus* all matched exactly within the conspecific representatives from the wide areas of western Palearctic. An exception is the *F.californica* which formed a unique BIN BOLD:AEJ3761 with 3.06% divergence from NN BIN BOLD:AAE6642 (includes specimens under the name of *F.fragilis* (synonym of *F.califonica*)).

The freshwater clams (family Sphaeriidae) are a cosmopolitan group inhabiting all types of freshwater habitats (Korniushin 2002, Rassam et al. 2020). The taxonomy and distribution of freshwater clams still need substantial clarification (Rassam et al. 2021). This is mainly because of limitations in diagnostically important morphological characters (Korniushin 2000, Voode 2017). From the South Caucasus region, a number of species are thought to belong to the genera *Sphaerium*, *Musculium* and *Euglesa*. The former two genera are represented with single species (*M.lacustre* and *S.corneum*), while the latter genera is represented with by least seven species (Zhadin 1952, Akramowski 1976). From these genera, we were able to obtain DNA barcodes for several taxa identified as *S.corneum*, *M.lacustre*, *E.casertana* and *E.subtruncata*. Three specimens of *M.lacustre* were matched with a specimen from Spain (BIN BOLD:AEE5622) with the maximum intra-BIN divergence of 0.36%. The NN BIN (with 1.6% divergence) is also represented with the COI haplotype of *M.lacustre* specimens from Europe. In contrast, COI barcodes for morphologically identified specimens as *S.corneum* were matched with single specimens of *S.nucleus* from the United Kingdom (BIN: BOLD:ACQ8004). Within this BIN, only the sequence was available before which, with our three additional sequences, resulted in a within-BIN maximum p-distance of 1.47%. The NN (with 3.85% divergence) BIN is BOLD:ABU6190 comprising *S.nucleus* specimens from central Europe, which are, on their own, closely-related (2.87% divergence) to *S.corneum* (BOLD:ADF3777) from central and south-west Europe. *S.nucleus* was usually considered an intraspecific variety of *S.corneum* (Piechocki 1989). However, according to Korniushin (2001) and Petkevičiūtė et al. (2018), there are several stable morphological and anatomical characteristics and, even more importantly, substantial genetic evidence that these two species are sister taxa. Due to observed genetic differences of our specimens to the corneum/nucleus group, it is worthwhile to investigate the South Caucasian *Sphaerium* representatives in more detail including multilocus phylogeny and morphology to solve its taxonomic affinities.

Another genus of clams with a complicated genetic structure is *Euglesa*. Specimens submitted to a barcoding pipeline were morphologically identified as either *E.casertana* (five specimens) or *E.subtruncata* (two specimens). The only specimen of putative *E.subtruncata* was validated under BIN: BOLD:ACQ3092, while the rest of the specimens formed unique genetic clusters with no clear systematic position. As an example, BIN BOLD:ACQ7011 contains specimens from Greece, Albania, Germany and one specimen from Georgia with a maximum intra-specific divergence of 1.71%. The closest NN BIN BOLD:AAG0350 (an unnamed clade) diverged with 1.92%. The remaining five specimens all turned out to belong to a yet unknown species under BINs BOLD:AEN6788 (5.13% divergence to NN) and BOLD:AEN0712 (3.8% divergence to NN BIN). Similar to *Sphaerium*, this genus is also difficult to classify, based on shell morphology alone due to limitations in taxonomically meaningful characters (Korniushin and Glaubrecht 2006, Clewing et al. 2013, Voode 2017, Rassam et al. 2021). Accordingly, a more detailed study is necessary to solve species-level taxonomy and even to validate the taxonomic value of currently-used identification (morphological) characters for the species-level classification of *Euglesa*.

One more specious family in the study area is the bivalve family Unionidae that includes at least five valid species occurring in South Caucasus, including *Uniocrassus*, *U.tumidus*, *U.pictorum*, *Anodontacygnea* and *A.anatina* (Graf 2007). In the present study, we sequenced representative specimens for all five species that perfectly matched with the conspecific barcodes from the BOLD system (Table 1). Similarly, specimens of other seven freshwater mollusc families, represented by a single species in the South Caucasus including *Acroloxuslacustris* (Acroloxidae), *Physellaacuta* (Physidae), *Bithyniatentaculata* (Bithyniidae), *Viviparuscostae* (Viviparidae), *Melanopsismingrelica* (Melanopsidae), *Theodoxusfluviatilis* (Neritidae) and one bivalve species *Corbiculafluminalis* (Cyrenidae) also formed unambiguous barcode clusters matching the conspecific sequences originated outside the study area.

## Conclusions

Our results clearly showed the insufficiency of the current knowledge of freshwater molluscs diversity in the South Caucasus region. In spite of the limited taxon coverage, nearly half of the studied taxa turned out to be in need of substantial taxonomic investigation/revision. In particular, nearly all genera with more than one known species are represented with regionally unique radiation and the species level taxonomy is inadequate. The South Caucasus region is considered a Plio-Pleistocene refugium and occurrence of unique or endemic lineages are not a surprise. However, a good understanding of its biodiversity is necessary to apply effective monitoring and conservation measures. In addition, the knowledge of the origin and phylogeography of most of the South Caucasian freshwater molluscs are generally missing (but see rare exception by Sands et al. 2019). Thus, obtained barcode data could pave the way to make further progress in this direction. A group of freshwater molluscs that were not investigated in the current project includes the representatives of the family Hydrobiidae – minute prosobranch snails. Only recently, this group turned out to be very species-rich in the South Caucasus (particularly in Georgia) (see, for instance, Grego et al. 2020). Although the systematics of this family in the South Caucasus is being studied by means of integrative approaches, still no quality barcodes are available for any of the species. Thus, diverse Hydrobiidae and some other freshwater mollusc families, for which only a sample of representatives have been studied until now, need to be further investigated in order to develop a useful barcode library. This particularly concerns the integrative taxonomic investigations to solve taxonomic ambiguities and clarify species-level diversity in the region.

## Supplementary Material

3037640C-08C5-5147-BCEB-DEA2D500DA73 10.3897/BDJ.10.e84887.suppl1 Supplementary material 1 Supplementary Table S1 Data type occurrences, sample data Brief description Details on barcoded freshwater molluscs from Georgia, Armenia and Azerbaijan. File: oo_709542.xlsx https://binary.pensoft.net/file/709542 Bikashvili Ani; Kachlishvili Nino; Japoshvili Bella; Mumladze Levan

## Figures and Tables

**Table 1.** BOLD summary data of barcoded Freshwater Molluscs with mean and maximum intraspecific and nearest neighbour (K2P) distances. Country Codes: AT = Austria, ALB = Albania, ARG = Argentina, AZR = Azerbaijan, ARM = Armenia, ALB = Albania, AU = Australia, BY = Belarus, BG = Bulgaria, BIH = Bosnia and Herzegovina, CH = China, CO = Colombia, CU = Cuba, CA = Canada, HR = Croatia, CZ = Czech Republic, ECUA = Ecuador, FI = Finland, FR = France, DE = Germany, GE = Georgia, GR = Greece, HU = Hungary, IT = Italy, IR = Iran, IQ = Iraq, IN = India, JP = Japan, KZ = Kazakhstan, KE = Kenya, LT = Lithuania, MA = Morocco, MX = Mexico, MLO = Moldova, ME = Montenegro, MT = Malta, MY - Malaysia, MM = Myanmar, NZ = New Zeland, NP = Nepal, NL = Netherlands, MK = North Macedonia, PL = Poland, PT = Portugal, PE = Peru, RU = Russia, RO = Romania, RS = Serbia, SE = Sweden, SI = Slovenia, SK = Slovakia, ESP = Spain, CH = Switzerland, SG = Singapore, TH = Thailand, TR = Turkey, UKR = Ukraine, UK = United Kingdom, UZB = Uzbekistan, US = United States VE = Venezuela. n = BIN member count.

Species	BIN	n	MeanISD	MaxISD	Country	Nearest BIN/ species	Distance to NN
*Ancylus* sp. 1	BOLD:AEN7656	12	0.19	0.55	GE	BOLD:AAD2028	4.95
*Ancylus* sp. 2	BOLD:AAD2028	185	1.49	3.39	TR, GR, MK, SI, IT, RO, ALB, GE, ME, AT, FR, ARM, AZR	BOLD:ACZ3241	3.3
*Bathyomphaluscontortus*	BOLD:AAK0034	20	0.75	1.61	DE, NL, AT, PL, GE	BOLD:ADR9065	9.45
*Gyraulusalbus*	BOLD:AAN4112	19	1.16	3.02	DE, ME, AT, PL, RS, CZ, GE	BOLD:AEB5660	7.55
*Segmentina* sp.	BOLD:AEN3217	3	0.22	0.32	GE	BOLD:AAN3912	11.89
*Anisus* sp.	BOLD:AEC8114	6	0.43	0.81	GE	BOLD:AAR3430	8.58
*Planorbisplanorbis*	BOLD:AED0778	5	0.39	0.97	GE	BOLD:ACS1294	3.4
*Planorbisplanorbis*	BOLD:ADJ5964	4	0.28	0.5	IR, GE	BOLD:ACS1294	2.1
*Ferrissiacalifornica*	BOLD:AEJ3761	3	0	0	GE	BOLD:AAE6642	3.06
*Ampullaceana* sp.	BOLD:AEN6567	2	0	0	GE	BOLD:ACI0501	4.97
*Radixeuphratica*	BOLD:ADJ8863	53	1.34	2.96	IQ, IR, GE, USB, RU	BOLD:AEI7975	2.82
*Radixeuphratica*	BOLD:ADK5204	5	0.96	1.7	IQ, GE	BOLD:ADJ8863	3.37
*Radixeuphratica*	BOLD:ADK6106	3	0.32	0.48	IQ, GE	BOLD:ADR3052	1.92
*Radixeuphratica*	BOLD:ADR3052	3	0.11	0.16	IQ, GE	BOLD:ADK6106	1.92
*Radixauricularia*	BOLD:ACI2007	14	0.46	0.84	ARM, GE	BOLD:AAD6712	2.88
*Radixauricularia*	BOLD:AAD6712	153	0.91	2.99	DE, PL, ME, HR, GR, MK, RU, ARM, CA, FR, ESP, CH, AT, US, GE	BOLD:ACI2007	2.88
*Peregrianaperegra*	BOLD:AAD0368	74	2.03	4.92	ALB, FR, RS, GR, MK, ME, DE, SK, RU, AT, IR, GE	BOLD:AEN6567	10.14
*Lymnaeastagnalis*	BOLD:AEM9638	6	0	0	GE	BOLD:ACQ2679	2.12
*Lymnaeastagnalis*	BOLD:AEN6037	9	0.73	1.4	GE, DE	BOLD:ACQ0092	2.43
*Galbatruncatula*	BOLD:ABA2623	50	0.99	2.74	FR, VE, IR, NP, SI, GR, RU, ME, ALB, GE	BOLD:AAI7214	4.03
*Galbaschirazensis*	BOLD:AAY4012	64	0.42	0.02	CA, VE, PE, ECUA, MX, IR, FR, US, CO, JP, GE	BOLD:ADR2784	7.84
*Stagnicola* sp.	BOLD:AEN6388	2	0.16	0.16	GE	BOLD:ACV7473	4.83
*Acroloxuslacustris*	BOLD:AAS0589	29	1.44	2.92	DE, TR, MK,GR, RS, AT, ALB, UKR	BOLD:ADK8211	2.9
*Physellaacuta*	BOLD:AAB6433	50	0.67	3.86	FR, US, GR, MK, IR, JP, MT, UKR, AZR, GE	BOLD:AEM0595	2.03
*Physellaacuta*	BOLD:AEM0595	358	1.72	6.35	US, FR, NL, CU, AU, CA, IN, ARG, GR, MK, TH, SG, MY, NZ, MM, IR, CN, JP, AT, IQ, KE, ESP, MT, ME, DE, UKR, AZR, PE, GE	BOLD:AAB6433	2.03
*Viviparuscostae*	BOLD:AEE7831	4	0.67	1.33	GE	BOLD:ADI2641	0.44
*Bithyniatentaculata*	BOLD:AAN3084	55	1.32	3.73	DE, US, AT, GE, RU, KZ, BY, UKR, RO	BOLD:AAF5645	7.77
*Melanopsismingrelica*	BOLD:AEB5510	4	0.16	0.32	GE	BOLD:AEB0981	3.85
*Theodoxusfluviatilis*	BOLD:A AA7898	291	1.8	7.25	DE, FI, AT, HR, HU, BIH, UKR, ME, ALB, MK,GR, RU, TR, BG, GE, MLD, FR, RO, PT, ESP, LT, GB, MA, IT, SK	BOLD:ACF4500	5.08
*Corbiculafluminalis*	BOLD:ACF4380	64	0.15	3.07	FR, ARG, HU, IN, RU, GE, AZR	BOLD:ACF5867	1.92
*Anodontaanatina*	BOLD:AAB7495	897	1.93	5	PO, SE, PT, IT, ESP, FR, HR, RU, HU, CZ, UKR, AT, BG, MA, TR, DE, KZ, GE	BOLD:AAF6127	10.81
*Anodontacygnea*	BOLD:AAF0516	110	0.28	2.1	SE, PT, DE, PL, FR, IT, CZ, GB, HU, AT, TR, RU, GE	BOLD:AEE8900	8.73
*Uniocrassus*	BOLD:AAF5083	175	0.52	2.17	AT, UKR, TR, DE, GE	BOLD:ADR4461	2.28
*Uniopictorum*	BOLD:AAD9208	232	0.36	2.68	AT, PL, GB, UKR, RU, IR, GR, SK, FR, TR, DE, GE, MLD	BOLD:ADR3328	2.38
*Uniotumidus*	BOLD:AAF0052	78	0.22	1.28	SE, PL, UKR, AT, DE, GB, RU, SK, GE, MLD	BOLD:ADR6944	9.39
*Sphaerium* sp.	BOLD:ACQ8004	4	0.73	1.47	GE, GB	BOLD:ABU6190	3.85
*Musculiumlacustre*	BOLD:AEE5622	4	0.18	0.36	ESP, GE	BOLD:ACQ4690	1.6
*Euglesa* sp. 1	BOLD:AEN6788	3	1.12	1.44	GE	BOLD:ACQ0055	5.13
*Euglesa* sp. 2	BOLD:AEN0712	1	N/A	N/A	GE	BOLD:ACQ0055	3.08
*Euglesa* sp. 3	BOLD:ACQ7011	4	1.03	1.71	ALB, GR, DE, GE	BOLD:AAG0350	1.92
*Euglesasubtruncata*	BOLD:ACQ3092	7	0.77	1.61	GE, IT, MK, AT, US	BOLD:ACQ6136	3.09
